# Misconceptions and health-seeking behavior among diabetic patients regarding foot ulcers: the hidden role of circulatory awareness in preventing amputation

**DOI:** 10.3389/fpubh.2026.1924054

**Published:** 2026-09-11

**Authors:** Abdulaziz Fahd AlKaabba, Yazeed AlHarbi, Fayez Zaben Al-Mutairi, Fawaz Sulaiman Alshaya, Omer Fahd bin Jassas, Faris Saleh Alkahtani, Mohammed Abdullah Al-Subaie, Faisal Mohammed Alaswad, Abdulrahman AlOtaibi, Abdulrahman Omar Almesned, Rahaf Naji Almutairi

**Affiliations:** Department of Family and Community Medicine, College of Medicine, Imam Mohammad Ibn Saud Islamic University (IMSIU), Riyadh, Saudi Arabia

**Keywords:** amputation prevention, diabetic foot ulcers, health-seeking behavior, misconceptions, Saudi Arabia

## Abstract

**Introduction:**

Diabetic foot ulcers (DFUs) are a leading cause of preventable amputation worldwide. Patient misconceptions and poor circulatory awareness are underrecognized drivers of delayed presentation in Saudi Arabia. This study aimed to assess DFU-related misconceptions, circulatory awareness, and health-seeking behavior, and identify predictors of delayed care-seeking.

**Methods:**

A cross-sectional study was conducted between February and April 2026 among 574 adult diabetic patients attending 11 outpatient facilities in Riyadh, Saudi Arabia. A validated structured questionnaire assessed misconceptions, circulation awareness, health-seeking behavior, and prior education sources. Descriptive statistics, *t*-tests, ANOVA, chi-square, Pearson correlation, and binary logistic regression were applied; significance was set at *p* < 0.05.

**Results:**

The mean misconception score was 3.04 ± 0.34 (scale 1–5); 55.6% indicated a wound warrants attention only when painful, 65.0% stated they would delay care for a painless blister, and 50.7% would wait for pain before treating a painless ulcer. Circulation awareness averaged 3.25 ± 0.38; only 28.4% adequately distinguished nerve damage from circulatory impairment. Only 45.6% had received structured foot care education, while internet or social media (61.9%) outranked healthcare providers (35.9%) as the primary information source. Male sex (OR = 1.536, 95% CI 1.092–2.161, *p* = 0.014) and longer diabetes duration (OR = 1.258, 95% CI 1.049–1.509, *p* = 0.014) were the only independent predictors of high delay propensity.

**Conclusion:**

Saudi diabetic patients demonstrated substantial DFU misconceptions and delay propensity, with pain-centric beliefs and inadequate formal education as key contributing factors. Structured, circulation-focused patient education within primary and family medicine settings may help address delayed presentation and support amputation prevention efforts.

## 1 Introduction

Diabetes mellitus (DM) constitutes one of the foremost global public health challenges of the twenty-first century. As of 2021, approximately 537 million adults worldwide were living with diabetes, with projections forecasting a rise to 783 million by 2045 (1). The Middle East and North Africa (MENA) region carries a disproportionately high burden, with Saudi Arabia consistently ranking among the nations with the highest diabetes prevalence globally, estimated at 18.3% in 2020 and rising (1, 2).

Among the most devastating sequelae of diabetes is the development of DFUs. Globally, a DFU occurs approximately every 20 s, and the lifetime risk ranges from 19 to 34% (3, 4). DFUs account for more than 60% of non-traumatic lower-extremity amputations worldwide and are associated with five-year mortality rates reaching 50–70% following initial amputation (4–6). In Saudi Arabia, the prevalence of DFUs has been reported across a range of 2.05 to 35.1%, reflecting variability in study populations and healthcare settings (7).

The pathophysiological triad underpinning DFU development encompasses peripheral sensory neuropathy, peripheral arterial disease (PAD), and altered immune response, each of which independently and synergistically impairs wound healing (8, 9). Crucially, peripheral neuropathy ablates pain sensation—the very symptom most patients rely upon to gauge wound severity. This pathological painlessness creates a paradoxical epidemiological trap: the patients most at risk of serious ulceration are the least likely to perceive urgency in seeking care (10, 11).

Beyond the biological dimension, delayed care-seeking behavior represents a critical and modifiable determinant of DFU outcomes. Studies from Saudi Arabia and the broader MENA region have consistently documented that a substantial proportion of patients present to clinicians only when wounds have progressed to advanced stages (12, 13). Misconceptions regarding the role of pain, the capacity of wounds to self-heal, the adequacy of home remedies, and the preventability of amputation have each been identified as drivers of this delay (14, 15).

A particular gap in existing research concerns the role of circulatory awareness. Understanding that impaired blood flow—independent of pain—can render even visually unremarkable wounds life-threatening is a core competency for appropriate self-monitoring in diabetic patients. Yet the conceptual distinction between neuropathic and ischemic ulceration pathways remains poorly understood even among patients with established diabetes (14). Healthcare education programs have traditionally focused on generic foot hygiene and wound inspection, leaving circulatory pathophysiology largely unaddressed in patient-facing educational materials (16).

In the Saudi context, prior cross-sectional studies have documented knowledge and practice gaps in diabetic foot care (17–19); however, fewer studies have specifically targeted the intersection of misconceptions, circulatory awareness, and health-seeking delay as an integrated construct. The evolving information landscape—characterized by over 95% smartphone penetration in Saudi Arabia and the widespread consumption of unverified social media health content—introduces a further dimension that warrants investigation (20).

The present study was therefore designed to assess the prevalence and patterns of DFU-related misconceptions, circulatory awareness, and health-seeking delay propensity among adult diabetic patients in Saudi Arabia, and to identify demographic and clinical predictors of delayed care-seeking. Findings are intended to inform targeted patient education strategies aimed at reducing preventable amputations.

## 2 Methods

### 2.1 Study design and setting

A cross-sectional, quantitative study design was employed. The study was conducted between February 8 and April 12, 2026, among adult diabetic patients attending 11 outpatient facilities in Riyadh, Saudi Arabia — specifically, five outpatient diabetic clinics and six family medicine centers. Eligible patients were identified consecutively at each facility during routine clinic visits. To ensure proportional representation across sites, the target sample was distributed across all 11 centers based on each facility’s average monthly diabetic patient census, and data collection proceeded simultaneously across all sites throughout the recruitment period. This study was conducted in accordance with the principles of the Declaration of Helsinki. Ethical approval was obtained from the Research Assistant Company on Research and Studies (Committee Registration No. HAP-01-R-146), with IRB Approval Number 00209.02/2026, granted on February 9, 2026. All participants provided written informed consent prior to enrollment, and participation was voluntary.

### 2.2 Participants

Eligible participants were adults aged 18 years or older with a confirmed diagnosis of diabetes mellitus (Type 1 or Type 2), who were capable of reading and completing the questionnaire. Although the original protocol specified a minimum disease duration of 1 year, participants who reported a duration of less than 1 year at the time of data collection were retained in the final sample, as their questionnaire responses were complete and valid. This group (*n* = 52, 9.1%) was therefore included in descriptive analyses; their presence reflects the reality of recruitment in active outpatient settings where diagnosis dates may be approximate or recently confirmed at the time of enrollment. Individuals with documented cognitive impairment, severe acute illness requiring emergency attention at the time of recruitment, or inability to provide informed consent were excluded. Consecutive eligible patients were recruited using convenience sampling during routine clinic visits.

Sample size was calculated using a single-proportion formula for cross-sectional studies, with a conservative 50% prevalence estimate for the primary outcome, a 95% confidence interval, and a 5% margin of error, yielding a minimum required sample of approximately 384 participants. The target was inflated by 15–20% to account for incomplete responses and to ensure adequate statistical power for binary logistic regression. Of 624 patients approached across all 11 sites during the recruitment period, 594 agreed to participate and returned completed questionnaires. Twenty of these were collected during the pilot-testing phase and were excluded from the final analysis, yielding a final analyzed sample of 574 participants (response rate: 92.0%). Of the 574 participants, 52 (9.1%) indicated they did not know their diabetes type. This was accepted as a valid response reflecting genuine uncertainty among patients, a pattern documented in other regional diabetic populations where patients are managed primarily by general practitioners without subspecialty labeling of their diagnosis. These participants were included in all general analyses but excluded from diabetes-type-specific subgroup comparisons.

### 2.3 Data collection instrument

A structured, self-administered questionnaire was developed in Arabic through a process of adaptation, cultural contextualization, and expert validation. Items were drawn and modified from validated instruments used in Saudi Arabian and international DFU knowledge and practice studies (5, 13–15), with language adapted to reflect the health literacy level and cultural context of the target Saudi outpatient population. The instrument comprised 34 items organized across 5 domains: 10 sociodemographic and clinical items (Section A), 12 Likert-scale misconception items (Section B), nine Likert-scale circulatory awareness items (Section C), nine Likert-scale health-seeking behavior items (Section D), and four categorical items on prior education and information sources (Section E).

Content validity was assessed through expert review by three specialists in family medicine, diabetic foot management, and patient education, each of whom independently reviewed all items for relevance, clarity, and cultural appropriateness. Feedback from this review led to minor rewording of six items to improve comprehension. The instrument was subsequently pilot-tested among 20 diabetic patients attending one of the participating clinics, who were not included in the final analysis. Pilot participants completed the questionnaire independently and were then interviewed briefly to identify any items that caused confusion or misinterpretation. No major structural changes were required following the pilot; two items received minor phrasing adjustments based on participant feedback. The finalized instrument was self-administered in Arabic, and no translation from another language was required, as the questionnaire was developed directly in Arabic for this population.

Participants were asked to report basic background information in the opening section, covering age group, sex, marital and employment status, highest level of education completed, diabetes type and how long they had lived with the condition, their current treatment, any prior foot problems, and how often they checked their own feet.

The next section examined how participants thought about foot wounds and their consequences, using 12 statements scored from 1 to 5, where 1 meant strong disagreement and 5 meant strong agreement—a higher total pointing to more misconceptions. The statements covered a range of potentially harmful beliefs: whether pain signals a wound worth worrying about, whether wounds can be left to resolve on their own, whether traditional or home-based treatments are sufficient, whether being able to walk normally means a wound is safe, and whether amputation is an unavoidable outcome regardless of what one does.

Nine statements on the same 1-to-5 scale then probed how well participants understood the role of blood circulation in foot health, with higher scores here reflecting stronger awareness rather than greater misconception. Items examined understanding of the relationship between impaired circulation and wound healing, the vascular risks of smoking, recognition of neuropathic symptoms, the importance of pulse monitoring, and the conceptual distinction between nerve damage and ischemic disease.

The fourth domain measured health-seeking behavior and delay propensity using nine items presenting hypothetical clinical scenarios. Four items reflected proactive care-seeking behavior and were reverse-coded prior to composite scoring, such that higher composite scores consistently indicated greater delay propensity across all items.

The fifth domain collected information on prior diabetic foot care education, the professional cadre who delivered that education, self-rated knowledge of foot care, and primary sources of foot health information.

Prior to full deployment, the instrument was pilot-tested among 20 diabetic patients who were not included in the final analysis. Content validity was assessed through expert review by three specialists in family medicine, diabetic foot management, and patient education. The complete instrument is provided as a Supplementary material.

### 2.4 Statistical analysis

Data were exported to IBM SPSS Statistics version 26.0 for analysis. Descriptive statistics—frequencies, percentages, means, and standard deviations—were calculated for all variables. Composite domain scores were computed as mean item scores within each domain. Cronbach’s alpha was computed for the misconception and circulation awareness composites and is reported descriptively for transparency (*α* = 0.005 and *α* = 0.041, respectively). These low values are acknowledged and reflect the intentional multi-domain structure of both scales, wherein items were designed to sample across several conceptually distinct belief subdomains — including pain-based reasoning, wound self-healing beliefs, home remedy reliance, ambulatory safety heuristics, and fatalistic attitudes toward amputation — rather than to measure a single latent trait. Cronbach’s alpha is an inappropriate reliability metric for such heterogeneous multi-domain instruments, as it assumes one-dimensionality; low alpha in this context signals item diversity rather than poor measurement quality. Mean composite scores are reported as summary descriptors of domain-level burden across these heterogeneous items. Future research should apply exploratory or confirmatory factor analysis to identify coherent subscale structures with improved internal consistency. Independent samples *t*-tests compared misconception and delay scores by sex, diabetes type, and prior DFU education receipt. One-way ANOVA compared scores across age groups, educational levels, and diabetes duration categories. Pearson’s correlation coefficient examined bivariate associations among the three composite scores. Binary logistic regression was performed with high delay propensity as the dependent variable, operationalized as an above-median composite delay score (median = 3.33). Median dichotomization was employed to enable identification of a clinically meaningful high-delay subgroup and to facilitate the use of binary logistic regression, which allows direct estimation of odds ratios and confidence intervals for each predictor — outputs with greater clinical interpretability than regression coefficients from a continuous outcome model. This approach has been used in comparable health-seeking behavior studies in diabetic populations. The median was preferred over an arbitrary threshold to ensure the cut-point was empirically grounded in the distribution of the present sample. Independent variables were entered simultaneously and included sex, age group, educational level, diabetes duration, misconception composite score, circulation awareness score, prior DFU education receipt, and foot self-examination frequency. All tests were two-tailed, and statistical significance was set at *p* < 0.05. For the binary logistic regression model, goodness-of-fit was assessed using the Hosmer-Lemeshow test (χ^2^ = 6.904, df = 6, *p* = 0.330), indicating acceptable model fit. Discriminatory ability was evaluated using the area under the receiver operating characteristic curve (AUC-ROC = 0.590, 95% CI 0.549–0.639), reflecting modest predictive performance consistent with a model examining attitudinal and behavioral predictors of a complex multi-determined outcome. Multicollinearity among independent variables was examined using variance inflation factors (VIF) computed on standardized continuous predictors; all VIF values were below 8.0, with no evidence of problematic collinearity. Variable coding for logistic regression was as follows: sex was coded as male = 1, female = 0; age group, educational level, diabetes duration, and foot self-examination frequency were entered as ordinal variables; misconception and circulation awareness composite scores were entered as standardized continuous variables; and prior DFU education receipt was coded as yes = 1, no or unknown = 0.

## 3 Results

### 3.1 Sociodemographic and clinical profile

The sociodemographic and clinical characteristics of the 574 study participants are presented in Table 1. The majority were male (54.4%, *n* = 312) and married (72.8%, *n* = 418). Age distribution was skewed toward older adulthood, with 44.8% (*n* = 257) in the uppermost age bracket and 26.8% (*n* = 154) aged 31–35 years. University education was the most prevalent attainment level (46.7%, *n* = 268), and more than half of participants were employed (50.3%, *n* = 289).

**Table 1:** Sociodemographic and clinical characteristics of study participants (*N* = 574).

Characteristics		*N*	%
Age group	20–25 years	43	7.5
	26–30 years	120	20.9
	31–35 years	154	26.8
	Above 40 years	257	44.8
Sex	Male	312	54.4
	Female	262	45.6
Marital status	Married	418	72.8
	Single	102	17.8
	Divorced	36	6.3
	Widowed	18	3.1
Educational attainment	Secondary	105	18.3
	Diploma	109	19.0
	University	268	46.7
	Master’s Degree	75	13.1
	Doctorate Degree	17	3.0
Employment status	Employed	289	50.3
	Homemaker	97	16.9
	Student	84	14.6
	Unemployed	59	10.3
	Retired	45	7.8
Type of diabetes	Type 1	108	18.8
	Type 2	414	72.1
	Unknown	52	9.1
Duration of diabetes	< 1 year	52	9.1
	1–5 years	169	29.4
	6–10 years	200	34.8
	> 10 years	153	26.7
Current diabetes treatment	Oral medication + Insulin	163	28.4
	Insulin only	158	27.5
	Oral medication only	156	27.2
	Diet + Oral medication	49	8.5
	Diet only	39	6.8
	Diet + Oral + Insulin	9	1.6
Previous foot problems	None	115	20.0
	Corns / calluses	124	21.6
	Numbness / tingling	112	19.5
	Blisters	83	14.5
	Corns + Numbness/tingling	65	11.3
	Healed ulcer	37	6.4
	Blisters + Numbness/tingling	21	3.7
	Current ulcer	11	1.9
	Previous amputation	6	1.0
Foot self-examination frequency	Daily	88	15.3
	Several times per week	119	20.7
	Weekly	127	22.1
	Monthly	146	25.4
	Never	94	16.4

The questionnaire combined participants aged 36–40 years and those above 40 into a single category. Percentages may not sum to 100% due to rounding.

Type 2 diabetes mellitus predominated (72.1%, *n* = 414), followed by Type 1 (18.8%, *n* = 108). The most common disease duration was 6–10 years since diagnosis (34.8%, *n* = 200). Combination oral medication and insulin was the most frequent treatment regimen (28.4%, *n* = 163), consistent with the distribution of diabetes type and disease duration across the sample. Regarding previous foot problems, corns or calluses were the most commonly reported condition (21.6%, *n* = 124), followed by numbness or tingling (19.5%, *n* = 112), and blisters (14.5%, *n* = 83). Composite neuropathic presentations — involving both corns or calluses and numbness or tingling — were reported by 11.3% (*n* = 65). A history of healed ulcer was present in 6.4% (*n* = 37), while current active ulceration and previous amputation were reported by 1.9% (*n* = 11) and 1.0% (*n* = 6), respectively. One in five participants (20.0%, *n* = 115) reported no prior foot problems. With respect to foot surveillance behavior, only 15.3% (*n* = 88) performed daily self-examination, while 16.4% (*n* = 94) reported never examining their feet; the remaining participants examined their feet weekly (22.1%), several times per week (20.7%), or monthly (25.4%).

One-way ANOVA revealed no statistically significant association between the type of previous foot problem reported and either delay propensity (*F* = 0.808, *p* = 0.581) or misconception burden (*F* = 1.868, *p* = 0.073), suggesting that clinical foot history alone did not substantially differentiate patients by their knowledge or behavioral profile in this sample. Similarly, no significant difference in delay propensity was detected between participants who reported neuropathic symptoms (mean = 3.29 ± 0.42) and those who did not (mean = 3.29 ± 0.41; *t* = 0.004, *p* = 0.997), nor between those with a history of ulceration or amputation and those without (mean = 3.24 vs. 3.29; *t* = −0.859, *p* = 0.391). These null findings underscore that delay propensity in this sample was not substantially determined by clinical foot history and is better explained by attitudinal and demographic factors, as elaborated in subsequent analyses.

### 3.2 Misconceptions about diabetic foot ulcers

Table 2 presents the item-level distribution and composite score for DFU-related misconceptions. The composite misconception score was 3.04 ± 0.34 (scale 1–5), indicating a moderate but clinically important burden across all 12 items. The most prevalent misconception was that a foot wound requires attention only when it becomes painful, endorsed by 55.6% of participants (mean = 3.43 ± 1.26). This was followed by the belief that normal ambulation confirms wound safety (49.8% agreement; mean = 3.28 ± 1.17) and the belief that a painless wound is usually not serious (51.2% agreement; mean = 3.26 ± 1.22).

**Table 2:** Misconception item-level response distributions and composite score (*N* = 574).

Statement	SD %	D %	N %	A %	SA %	Agree + SA %	Mean (SD)
1. Painless wound on the foot is usually not serious	8.9	22.8	17.1	36.1	15.2	51.2	3.26 (1.22)
2. Most foot wounds will heal without medical treatment	10.6	26.5	24.2	24.4	14.3	38.7	3.05 (1.23)
3. Home or traditional remedies are sufficient for treating foot wounds	8.9	30.0	20.2	29.3	11.7	40.9	3.05 (1.19)
4. A foot wound only requires attention when it becomes painful	8.4	18.5	17.6	32.6	23.0	55.6	3.43 (1.26)
5. Diabetic foot ulcers are caused mainly by poor hygiene	10.6	30.5	24.9	23.5	10.5	34.0	2.93 (1.17)
6. A foot wound is not dangerous as long as normal walking is maintained	8.0	20.0	22.1	35.4	14.5	49.8	3.28 (1.17)
7. Covering a wound and avoiding clinical review is preferable to seeking care	10.6	31.2	25.4	23.0	9.8	32.8	2.90 (1.16)
8. Amputation occurs suddenly and cannot be prevented by early treatment	11.8	30.3	22.0	24.6	11.3	35.9	2.93 (1.21)
9. A wound that appears dry and clean does not require medical review	7.3	26.0	22.5	28.6	15.7	44.3	3.19 (1.20)
10. Foot ulcers develop only in patients with very high blood glucose levels	9.6	29.3	28.2	21.8	11.1	32.9	2.96 (1.16)
11. Changing footwear alone is sufficient to treat a diabetic foot wound	12.0	35.2	28.0	16.0	8.7	24.7	2.74 (1.13)
12. A foot blister should be drained at home to release fluid	13.2	35.4	23.7	17.8	9.9	27.7	2.76 (1.18)
Composite Score							3.04 (0.34)

SD, Strongly Disagree; D, Disagree; N, Neutral; A, Agree; SA, Strongly Agree. Higher scores indicate a greater misconception burden. Composite score = mean of 12 items (range 1–5).

The lowest misconception prevalence was observed for the belief that changing footwear alone is sufficient treatment (24.7% agreement) and that a foot blister should be drained at home (27.7% agreement). Nevertheless, all 12 items attracted agreement from at least one quarter of participants, and the pain-centric belief cluster—spanning the perception that pain signals urgency, that normal walking confers safety, and that a painless ulcer warrants no action until pain develops—constituted the dominant and internally consistent pattern across the sample.

### 3.3 Circulatory awareness and peripheral vascular disease knowledge

Circulatory awareness item scores are summarized in Table 3. Blood circulation awareness averaged 3.25 ± 0.38 across the nine items, with responses spread unevenly rather than clustering around any single level. Two areas stood out as relatively better understood: roughly half of participants recognized that poor circulation slows down wound healing (52.4%; mean 3.38 ± 1.10), and a similar proportion connected foot numbness to possible nerve or circulatory trouble (50.5%; mean 3.40 ± 1.13).

**Table 3:** Circulation awareness item-level agreement and descriptive statistics (*N* = 574).

Statement	Agree + SA %	Mean (SD)
1. Poor circulation can make a small foot wound serious	50.0	3.25 (1.12)
2. A painless ulcer is still dangerous if circulation is poor	45.3	3.17 (1.15)
3. Smoking worsens circulation and increases ulcer risk	48.6	3.32 (1.12)
4. Numbness in the feet can signal nerve or circulatory problems	50.5	3.40 (1.13)
5. Checking foot pulses and blood flow is important in diabetes	45.8	3.30 (1.10)
6. Poor circulation slows wound healing in the feet	52.4	3.38 (1.10)
7. Early detection of circulatory problems can prevent amputation	47.7	3.27 (1.15)
8. I understand the difference between nerve damage and circulatory disease	28.4	2.89 (1.09)
9. Healthcare providers explained how circulation affects foot ulcer risk	45.3	3.22 (1.14)
Composite Circulation Awareness Score		3.25 (0.38)

Agree + SA = combined percentage of “Agree” and “Strongly Agree” responses. Higher scores reflect better circulatory awareness. Composite score = mean of 9 items (range 1–5).

Where participants struggled most was in separating nerve damage from ischemic circulatory disease—a distinction that only 28.4% could make, the weakest result across all 21 knowledge and misconception items combined. What made this finding particularly telling was the neutral response rate of 36.9%, suggesting that most non-agreeing participants were simply unsure rather than actively wrong—a meaningful difference from a teaching standpoint. Separately, fewer than half (45.3%) said their healthcare provider had ever walked them through how circulation specifically affects foot ulcer risk, pointing to a gap that sits squarely within the clinical encounter rather than the patient alone.

### 3.4 Health-seeking behavior and delay propensity

The composite delay score was 3.29 ± 0.41, indicating a predominantly delay-prone intended behavioral profile across the sample. In response to the scenario involving a small painless blister, 65.3% of participants stated they would wait several days before contacting a doctor (mean = 3.74 ± 1.14). Similarly, 50.7% indicated they would continue their daily activities and seek care only if such an ulcer eventually became painful, reflecting a consistent pattern of pain-contingent intended care-seeking.

Intended prompt care-seeking was consistently low across all proactive scenarios. Only 27.0% stated they would attend a clinic within 24 h of noticing redness or swelling around a foot wound, and an equal proportion (27.0%) indicated they would immediately discuss new-onset numbness or tingling with a doctor. Only 30.8% reported they would immediately attend a specialist diabetic-foot clinic if referred by a doctor. Most critically, when asked whether they would feel confident seeking medical attention early upon noticing any foot change, only 24.2% of participants indicated they were likely or very likely to do so. Of the remaining participants, 52.8% actively disagreed or strongly disagreed with this statement, while 23.0% responded neutrally. Taken together, 75.8% of participants either actively lacked confidence or could not affirm confidence in early care-seeking, representing the most significant self-efficacy gap identified across all items (Table 4).

**Table 4:** Health-seeking behavior and delay propensity item-level responses (*N* = 574).

Scenario	L + VL %	Mean (SD)	Item type
1. Would wait several days before contacting a doctor for a small painless blister	65.3	3.74 (1.14)	Delay
2. Would attend a clinic within 24 h of noticing redness or swelling around a foot wound	27.0	2.67 (1.23)	Prompt‡
3. Would try home remedies before visiting a doctor for a foot wound	42.3	3.18 (1.19)	Delay
4. Would still avoid the clinic due to time or cost concerns even if a wound did not improve	32.8	2.87 (1.17)	Delay
5. Would immediately discuss new-onset numbness or tingling in the feet with a doctor	27.0	2.70 (1.22)	Prompt‡
6. Would continue daily activities and seek treatment only if a painless ulcer became painful	50.7	3.34 (1.17)	Delay
7. Would immediately attend a specialist diabetic-foot clinic if referred	30.8	2.70 (1.28)	Prompt‡
8. Generally, tends to delay seeking care for foot problems until symptoms are severe	39.5	3.13 (1.13)	Delay
9. Feels confident about seeking medical attention early if any change is noticed§	24.2	2.60 (1.21)	Prompt‡
Composite Delay Score		3.29 (0.41)	

L, Likely; VL, Very Likely. ‡Prompt items were reverse-coded prior to composite delay scoring, such that all items contribute to the same direction: higher composite score = greater delay propensity. §Item 9 reflects self-efficacy for early care-seeking; the percentage reported (24.2%) represents those who felt confident (Likely or Very Likely). Composite delay score range 1–5.

### 3.5 Prior education, information sources, and inferential statistics

Of the 574 participants, 262 (45.6%) reported having ever received specific diabetic foot care education; 211 (36.8%) had never received it, and 101 (17.6%) could not recall. Among those who had received education (*n* = 262), physicians were the most frequently cited sole providers (*n* = 79, 30.2% of the educated subgroup), followed by nurses alone (*n* = 63, 24.0%) and diabetes educators alone (*n* = 39, 14.9%). When combination delivery was also considered, physicians were involved in education delivery in 54.6% of cases (sole or in combination), nurses in 44.3%, and diabetes educators in 31.7%. Pharmacists were cited as sole providers by only 4 participants (1.5%). Self-rated knowledge was predominantly average (235 participants, 40.9%), while 182 (31.7%) rated their knowledge as poor or very poor, and only 157 (27.3%) as good or excellent. Regarding information sources, internet or social media was the most frequently cited channel, mentioned either alone or in combination with other sources by approximately 61.9% of participants. Family members or friends were cited by approximately 41.2%, television or radio by approximately 30.1%, and healthcare providers by approximately 35.9%. Printed educational materials were cited by only 12 participants (2.1%), and 7 participants (1.2%) reported having received no information about diabetic foot care from any source. These figures are based on a select-all-that-apply response format; individual percentages therefore do not sum to 100%.

Pearson correlation analysis revealed no statistically significant association between the misconception composite score and the delay composite (*r* = 0.041, *p* = 0.328), nor between circulation awareness and delay (*r* = 0.001, *p* = 0.973). When diabetes duration was examined against delay propensity using one-way ANOVA, a statistically significant pattern emerged (*F* = 3.307, *p* = 0.020). Those who had been living with diabetes the longest tended to score highest on delay—participants past the 10-year mark averaged 3.33, closely followed by the 6–10-year group at 3.32. By contrast, those earlier in their diagnosis scored noticeably lower, with the 1–5-year group averaging 3.21 and those diagnosed under a year sitting at 3.24. Put simply, the longer someone had been managing the condition, the more likely they were to put off seeking care when a foot problem arose. No statistically significant differences were detected in misconception or delay scores by age group (*F* = 2.259, *p* = 0.081), educational attainment (*F* = 0.784, *p* = 0.536), diabetes type, sex, or prior DFU education receipt.

Binary logistic regression (Table 5) confirmed male sex (OR = 1.536, 95% CI 1.092–2.161, *p* = 0.014) and longer diabetes duration (OR = 1.258, 95% CI 1.049–1.509, *p* = 0.014) as the only independent predictors of high delay propensity. Misconception score, circulation awareness, educational level, age group, prior DFU education, and foot self-examination frequency were not significant independent predictors. The overall model was statistically significant (model chi-square = 16.247, df = 8, *p* = 0.039; McFadden pseudo-R^2^ = 0.021).

**Table 5:** Binary logistic regression: predictors of high delay propensity (*N* = 574).

Variable	OR	95% CI	*p*-value	Sig.
Male sex (vs. Female)	1.536	1.092–2.161	0.014	*
Age group	1.002	0.842–1.193	0.979	—
Educational level	0.894	0.756–1.057	0.189	—
Diabetes duration	1.258	1.049–1.509	0.014	*
Misconception composite score	1.194	0.731–1.953	0.479	—
Circulation awareness score	1.244	0.795–1.946	0.340	—
Prior DFU education received	1.100	0.782–1.546	0.585	—
Foot self-examination frequency	1.088	0.955–1.239	0.205	—

Dependent variable: high delay propensity (above-median composite delay score). OR, odds ratio; CI, confidence interval. * *p* < 0.05. Model chi-square = 16.247, df = 8, *p* = 0.039; McFadden pseudo-R^2^ = 0.021.

## 4 Discussion

The findings from this study point to a set of interconnected barriers — rooted in how patients perceive pain, understand circulatory risk, and navigate care decisions — that collectively sustain a pattern of delayed DFU presentation in Saudi Arabia. Notably, these barriers did not operate uniformly across the sample; sociodemographic and clinical characteristics, particularly sex and diabetes duration, emerged as significant independent determinants of delay propensity, while clinical foot history did not significantly differentiate patients by misconception burden or behavioral profile. Five major findings warrant discussion.

### 4.1 Predominance of pain-centric misconceptions

The most prevalent and clinically consequential pattern identified in this study was the dominance of pain-centric belief systems. More than half of participants (55.6%) indicated that a foot wound warrants attention only once it becomes painful, and 51.2% did not regard a painless wound as a serious concern. This reasoning is fundamentally incompatible with the pathophysiology of peripheral neuropathy, in which loss of protective sensation renders pain an unreliable indicator of wound severity. In patients with established sensory neuropathy, the same nerve damage responsible for producing an asymptomatic wound also eliminates the pain signal that would ordinarily prompt care-seeking, creating a physiological paradox in which the highest-risk wounds are the least likely to generate perceived urgency (10). The concurrent finding that 65.3% would delay seeking care for a painless blister, and that 50.7% would wait for pain to develop before treating a painless ulcer, demonstrates the behavioral downstream consequences of this belief system with a high degree of internal consistency across the measurement domains.

These findings align with those reported in a Saudi primary care cohort, where the belief that wounds require attention only upon becoming painful was identified as the most persistent barrier to early DFU presentation (12). A proposed mechanistic explanation is well supported in the literature: in patients with sensory neuropathy, the same nerve damage implicated in asymptomatic ulceration is also thought to eliminate the pain signal that would otherwise prompt care-seeking (10). These findings underscore the importance of patient education that explicitly addresses pain-contingent reasoning and redirects self-monitoring toward visual inspection, changes in wound appearance, and recognition of progressive neurological symptoms.

### 4.2 Circulatory awareness gaps and the neuropathy-ischemia distinction

While general awareness of the relationship between poor circulation and wound healing was moderate (composite score 3.25 ± 0.38), the most critically deficient area was the conceptual distinction between nerve damage and ischemic disease in the diabetic foot, with only 28.4% of participants demonstrating adequate understanding. The clinical consequences of this gap are not trivial. Neuropathic and ischemic ulcers differ substantially in their presentation, management priorities, and prognostic trajectories, and a patient unable to differentiate between the numbness of sensory neuropathy and the cold, pale, pulseless limb of arterial insufficiency may catastrophically misinterpret early ischemic warning signs as benign diabetic tingling (8, 9). The neutral response rate of 36.9% for this item suggests that uncertainty, rather than active misconception, is the primary obstacle—an observation with more optimistic educational implications, as uncertainty may be more amenable to targeted intervention than entrenched false beliefs.

More broadly, the absence of a statistically significant correlation between circulation awareness scores and delay propensity (r = 0.001, *p* = 0.973) suggests that general circulatory knowledge, at the composite level, was insufficient to translate into earlier intended care-seeking in this sample. This pattern is consistent with health behavior frameworks documenting the knowledge-intention-behavior gap, and supports a shift in educational priorities from information provision toward behavioral activation and action planning (21, 22). It is also worth noting that neither misconception burden nor circulation awareness score emerged as a significant independent predictor of delay in the logistic regression model, suggesting that the relationship between patient knowledge and behavioral intention in this domain may be more complex than a direct linear association, and may be substantially mediated by factors such as self-efficacy, sociocultural norms, and access to care.

### 4.3 Sex and diabetes duration as independent predictors of delay

Binary logistic regression identified male sex (OR = 1.536, 95% CI 1.092–2.161, *p* = 0.014) and longer diabetes duration (OR = 1.258, 95% CI 1.049–1.509, *p* = 0.014) as the only independent predictors of high delay propensity. Age group, educational level, prior foot care education receipt, misconception burden, circulation awareness, and foot self-examination frequency were not independently associated with delay once all variables were entered simultaneously.

The association between male sex and higher delay propensity may reflect, at least in part, sociocultural norms observed across Gulf populations, wherein men may be less inclined to seek medical attention until symptoms are perceived as functionally impairing — a pattern that has been noted in diabetic foot care studies conducted in the region (5, 13). It is hypothesized that educational messaging targeted at male patients may benefit from framing early care-seeking within contexts that resonate with occupational functioning and physical capability, though this hypothesis warrants direct testing in future intervention studies.

The association between longer diabetes duration and greater delay propensity is less straightforward and may plausibly reflect more than one contributing pathway. One proposed explanation is that patients with longer disease duration are more likely to have developed clinically significant peripheral neuropathy, which may reduce the perceived salience of early foot warning signs (10). A second hypothesis involves chronic disease normalization — the gradual adaptation to symptoms such as tingling, paresthesia, or thermal insensitivity as anticipated features of long-standing diabetes rather than as signals warranting clinical attention (23). These remain hypothetical pathways, and the cross-sectional design of this study precludes determination of the direction or causality of these associations. Nevertheless, the finding is consistent with a large United States national cohort study that similarly identified diabetes duration as a significant independent determinant of DFU prevention awareness (24), lending external validity to the observed association.

The absence of significant associations between clinical foot history — including the presence of neuropathic symptoms, prior ulceration, or amputation — and either delay propensity or misconception burden warrants comment. While it might be anticipated that patients with prior foot complications would demonstrate greater urgency in care-seeking, the data did not support this. Patients with a history of healed ulcer, current ulceration, or previous amputation did not demonstrate significantly lower delay scores than those with no prior foot problems (*p* = 0.391). This finding suggests that clinical experience with foot complications did not, in itself, reliably translate into more proactive intended behavior in this sample. Whether this reflects insufficient patient education following prior foot events, habituation to chronic foot pathology, or other factors cannot be determined from this study design, but it represents an important observation for clinical teams responsible for the follow-up of patients with established DFU history.

#### 4.3.1 Low specialist compliance and patient self-efficacy

Only 30.8% of participants indicated they would immediately attend a specialist diabetic-foot clinic if referred, and 52.8% explicitly reported lacking confidence in their capacity for early care-seeking. Self-efficacy — defined as an individual’s perceived capacity to execute a specific health-promoting behavior — is recognized as a central construct in the Health Belief Model and Social Cognitive Theory, and has been associated with stronger behavioral prediction than knowledge alone in multiple health behavior contexts (21, 22). The non-significant association between misconception scores and delay propensity (r = 0.041, *p* = 0.328) in the present study may partly reflect this mechanism: patients may hold correct beliefs yet still fail to act on them if they lack confidence in their own capacity to navigate the healthcare system promptly.

These findings suggest that clinical communication interventions targeting DFU delay should address not only what patients know, but whether patients perceive themselves as capable of and entitled to seek early care. Brief motivational interviewing, action planning — defined as the advance specification of when, where, and how one would seek care in response to a defined foot symptom — and proactive clarification of referral pathways have been proposed as potentially more effective than purely didactic educational approaches in comparable settings, though their direct efficacy in the Saudi diabetic foot context remains to be tested. Given that 75.8% of participants in this study either actively lacked or could not affirm confidence in early care-seeking, self-efficacy represents arguably the most modifiable attitudinal target identified across all domains assessed.

### 4.4 Education deficits and the digital information environment

The finding that fewer than half of participants (45.6%) had ever received structured foot care education, despite all being established outpatient diabetic patients, represents a major systems-level failure (16, 18, 25). The predominance of physician-delivered education, with comparatively limited involvement of nurses and diabetes educators, reflects the underutilization of allied health professionals who are well-positioned to deliver systematic, structured, and culturally sensitive foot care education at scale (16, 18, 26, 27).

Equally important is the information environment in which patients form their beliefs. Internet and social media outranked all formal healthcare sources as the primary information channel for diabetic foot health, cited by approximately 61.9% of participants compared with 35.9% who cited healthcare providers. Comparable patterns of internet and social media dominance as primary health information sources have been documented in other Saudi diabetic foot awareness studies (18, 20, 28), suggesting that unverified digital health content functions as a parallel educational system operating largely outside clinical oversight. Health authorities and diabetes care teams are encouraged to engage proactively with this landscape. The development and promotion of evidence-based, Arabic-language digital resources may represent a practical step toward offering patients access to verified information through channels they already use, with the potential to complement — though not replace — structured clinical education.

### 4.5 Limitations

Several limitations should be considered when interpreting these findings. First, the use of convenience sampling from 11 outpatient facilities affiliated with a single university in Riyadh limits the generalizability of findings to other regions of Saudi Arabia, rural populations, and patients managed in non-academic or primary care settings. Second, as an intention-based instrument measuring hypothetical behavioral responses to clinical scenarios rather than observed real-world behavior, all findings in the health-seeking behavior domain reflect stated intentions and are subject to social desirability bias; actual delay rates in clinical practice may be higher than those reported. Third, recall bias may have affected responses to questions on prior DFU education receipt and information source use, particularly given that 17.6% of participants could not recall whether they had received structured foot care education. Fourth, the cross-sectional design precludes determination of the temporal sequence or directionality of any observed associations; specifically, it cannot be established whether misconceptions preceded and contributed to delay behavior, or whether other unmeasured factors — including health system access, prior clinical encounters, and individual health literacy — contributed to both. Fifth, the low Cronbach’s alpha values for the misconception (*α* = 0.005) and circulation awareness (*α* = 0.041) composites indicate poor internal consistency, reflecting the intentional multi-domain item structure of both scales rather than a unidimensional measurement model. While mean composite scoring is reported as a descriptive summary, the psychometric limitations of these composites should be acknowledged, and future research should apply exploratory and confirmatory factor analysis to identify coherent subscale structures before composite scores are used as predictors in regression models. Sixth, median dichotomization of the delay composite score for logistic regression is an acknowledged limitation, as any threshold-based categorization of a continuous outcome reduces statistical power and introduces some degree of arbitrariness in group definition. Seventh, the overall logistic regression model demonstrated only modest discriminatory ability (AUC-ROC = 0.590), indicating that the predictors examined explain a limited proportion of variance in delay propensity, and that important unmeasured determinants — potentially including health system familiarity, social support, and prior clinical experiences — were not captured. Finally, the study relied entirely on self-reported measures; no objective clinical data on neuropathy severity, ankle-brachial index, wound status, or glycemic control were collected, precluding examination of biological pathways and limiting the clinical contextualization of behavioral findings.

## 5 Conclusion

Saudi adult diabetic patients demonstrated a substantial burden of DFU-related misconceptions, moderate but incomplete circulatory awareness, and a predominantly delay-prone intended health-seeking profile. Pain-centric beliefs constituted the single most pervasive misconception, with 55.6% indicating that wounds warrant attention only when painful and 65.3% stating they would delay care for a painless blister. Male sex (OR = 1.536, *p* = 0.014) and longer diabetes duration (OR = 1.258, *p* = 0.014) were the only independent predictors of high delay propensity, identifying two subgroups that may benefit from specifically tailored educational approaches.

These findings point to several priorities for patient education and clinical communication. Educational content may benefit from directly addressing pain-contingent reasoning, reorienting patients toward visual self-monitoring, symptom-independent foot inspection, and clearly defined criteria for seeking care — an approach that differs from general awareness messaging, which raises knowledge without necessarily prompting behavior change. Delivery of foot care education may also benefit from expansion beyond physician consultations to include nurses, diabetes educators, and pharmacists, who have repeated contact with diabetic patients across clinical encounters. Efforts to strengthen patient self-efficacy for early care-seeking appear warranted, given that 52.8% of participants actively lacked confidence in their ability to seek timely care. The development of validated, evidence-based, Arabic-language digital resources may help address the current dominance of unverified social media content, which was cited as the primary information source by 61.9% of this population. If addressed collectively, these gaps may contribute to reducing the burden of delayed DFU presentation in Saudi Arabia, though intervention studies are needed to confirm their effectiveness in improving care-seeking behavior and clinical outcomes.

## Data Availability

The original contributions presented in the study are included in the article/Supplementary material, further inquiries can be directed to the corresponding author.
