# Supplementary material for: Misconceptions and health-seeking behavior among diabetic patients regarding foot ulcers: the hidden role of circulatory awareness in preventing amputation
Source: Front Public Health. 2026 Sep 11;14:1924054. doi: 10.3389/fpubh.2026.1924054 (PMC13612442; doi:10.3389/fpubh.2026.1924054)
Supplement: Supplementary file 1 [file Data_Sheet_1.PDF]

Supplementary Material

1 Survey Questionnaire

This survey is part of a research study titled "Circulation-Related Misconceptions and Delayed Care for Diabetic Foot Ulcers: Implications for Amputation Prevention and Patient Education". Please answer honestly. There are no right or wrong answers.

Section A – Sociodemographic and Clinical Profile

Please tick (✓) the appropriate circle which corresponds to your answer.

1.1 Age

- 20-25 years
- 26-30 years
- 31-35 years
- 36-40 years
- Above 40 years

1.2 Sex

- Male
- Female

1.3 Marital Status

- Single
- Married
- Divorced
- Widowed

1.4 Highest Educational Attainment

- Secondary
- Diploma
- University
- Master Degree
- Doctorate Degree

1.5 Employment Status

- Employed
- Unemployed
- Retired
- Student
- Homemaker

1.6 Type of Diabetes

- Type 1

- Type 2
- I don’t know

1.7 Duration since first diagnosed?

- Less that 1 year
- 1-5 years
- 6-10 years
- More than 10 years

1.8 Current diabetes treatment (select all that apply)

- Diet only
- Oral medication
- Insulin
- Others specify

1.9 Previous foot problems (select all that apply)

- None
- Corns / calluses
- Blisters
- Numbness/tingling
- Healed ulcer
- Current ulcer
- Previous amputation

1.10 How often do you examine your feet yourself?

- Daily
- Several times per week
- Weekly
- Monthly
- Never

Section B – Misconceptions About Diabetic Foot Ulcers

Please tick (✓) the appropriate box which corresponds to your answer.
Scale: 1 = Strongly Disagree | 2 = Disagree | 3 = Neutral | 4 = Agree | 5 = Strongly Agree
(High score = more misconceptions)

INDICATORS	5	4	3	2	1
1. If a wound on my foot does not hurt, it is usually not serious.					
2. Most diabetic foot wounds will heal on their own without medical help.					
3. Traditional/home remedies are usually enough for treating diabetic foot wounds.					
4. I should only worry about a foot wound if it becomes painful.					

5. Diabetic foot ulcers are mainly caused by poor cleanliness rather than diabetes.					
6. As long as I can walk normally, a foot wound is not dangerous.					
7. Covering a wound tightly and ignoring it is better than visiting a clinic.					
8. Amputation usually happens suddenly and cannot be prevented by early treatment.					
9. If the wound looks dry and clean, there is no need to visit a doctor.					
10. Only people with very high blood sugar develop foot ulcers.					
11. Changing shoes alone is enough to treat a diabetic foot wound.					

Adapted from misconceptions identified in Saudi patients by Al-Balawi & Sheikh 2022 and in patients/relatives by Alsaigh et al. 2022

Section C – Circulation Awareness & Peripheral Vascular Disease

Please tick (✓) the appropriate box which corresponds to your answer.
Scale: 1 = Strongly Disagree | 2 = Disagree | 3 = Neutral | 4 = Agree | 5 = Strongly Agree
(High score = better awareness)

INDICATORS	5	4	3	2	1
13. Poor blood circulation in the legs can make a small foot wound become serious.					
14. A painless ulcer on the foot can still be dangerous if circulation is poor.					
15. Smoking can worsen circulation and increase the risk of ulcers.					
16. Numbness or loss of feeling in the feet can be a sign of nerve/circulation problems.					
17. Checking foot pulses and blood flow is important for people with diabetes.					
18. Poor circulation can slow wound healing in the feet.					
19. Early detection of circulation problems can help prevent amputation.					
20. I understand the difference between neuropathy and circulation problems.					
21. Healthcare providers have clearly explained how circulation affects diabetic-foot risk.					

Adapted from Based on vascular-risk awareness gaps identified by Jia et al. 2022 and Sarmiento-Luna et al. 2023

Section D – Health-Seeking Behavior & Delay Propensity

Please tick (✓) the appropriate box which corresponds to your answer. **Scale:** 1 = Very unlikely | 2 = Unlikely | 3 = Neutral | 4 = Likely | 5 = Very likely *(High score = worse)*

INDICATORS	5	4	3	2	1
22. If I saw a small painless blister, I would wait several days before contacting a doctor.					
23. If I saw redness/swelling around a foot cut, I would go to a clinic within 24					

hours.					
24. If I had a wound, I would try home remedies before visiting a doctor.					
25. If a wound didn't improve after a few days, I would still avoid going to the clinic due to time/cost concerns.					
26. If I felt new numbness or tingling, I would immediately discuss it with a doctor.					
27. If a painless ulcer appeared, I would continue daily activities and seek treatment only if it becomes painful.					
28. If advised to visit a specialized diabetic-foot clinic, I would do so immediately.					
Direct Self-Assessment Items					
29. In general, I tend to delay seeking medical care for foot problems until they become severe.					
30. I feel confident that I would seek medical attention early if I noticed any change in my feet. (reverse-coded)					

Adapted from Jia et al. 2022, Sarmiento-Luna et al. 2023, Al-Balawi & Sheikh, 2022, and Alsaigh et all., 2022

Section E – Previous Education & Information Sources

Please tick (✓) the appropriate box which corresponds to your answer.

31. Have you ever received specific education about diabetic foot care?
    ☐ Yes ☐ No ☐ I don't remember
32. If yes, who provided the education? (Select all that apply.)
    ☐ Doctor ☐ Nurse ☐ Diabetes educator ☐ Pharmacist ☐ Other: ____
33. How would you rate your overall knowledge of diabetic-foot care?
    ☐ Very poor ☐ Poor ☐ Average ☐ Good ☐ Excellent
34. Sources of information (select all that apply):
    ☐ Healthcare providers ☐ Family/friends
    ☐ Internet/social media ☐ TV/radio
    ☐ Printed brochures/posters ☐ I have not received any information about diabetic foot care

Adapted from Based on vascular-risk awareness gaps identified by Jia et al. 2022

Thank you for your participation!

2 References:

Jia H, Wang X, Cheng J. Knowledge, attitudes, and practices associated with diabetic foot prevention among rural adults with diabetes in North China. Front Public Health. (2022) 10:876105. doi:10.3389/fpubh.2022.876105

Albalawi MA, Sheikh WA. Knowledge, attitude and practice about foot care among adult diabetic patients at Prince Abdul-Aziz bin Majid Diabetes Center in Al Madinah City, Saudi Arabia. Egypt J Hosp Med. (2022) 87(1):1864-1871. doi: 10.21608/ejhm.2022.231637

Alsaigh SH, Alzaghran RH, Alahmari DA, Hameed LN, Alfurayh KM, Alaql KB, et al. Knowledge, awareness, and practice related to diabetic foot ulcer among healthcare workers and diabetic patients and their relatives in Saudi Arabia: a cross-sectional study. Cureus. (2022) 14(12):e32221. doi:10.7759/cureus.32221

Sarmiento-Luna FB, Nava-Navarro V, Baez-Hernandez FJ, Morales-Nieto A, Blanco-Alvarez VM. Validation of a knowledge and attitude instrument for nurses for the prevention of the diabetic foot. Enferm Glob. (2023) 22(70):101. doi:10.6018/eglobal.547181.
